# NRF2-Dependent Placental Effects Vary by Sex and Dose following Gestational Exposure to Ultrafine Particles

**DOI:** 10.3390/antiox11020352

**Published:** 2022-02-10

**Authors:** Jonathan C. Behlen, Carmen H. Lau, Drew Pendleton, Yixin Li, Aline Rodrigues Hoffmann, Michael C. Golding, Renyi Zhang, Natalie M. Johnson

**Affiliations:** 1 Department of Environmental and Occupational Health, Texas A&M University, College Station, TX 77843, USA; jbehlen@cvm.tamu.edu (J.C.B.); drew976@tamu.edu (D.P.); 2 Department of Veterinary Pathobiology, Texas A&M University, College Station, TX 77843, USA; clau@cvm.tamu.edu; 3 Department of Chemistry, Texas A&M University, College Station, TX 77843, USA; yxli11@tamu.edu (Y.L.); renyi-zhang@tamu.edu (R.Z.); 4 Department of Comparative, Diagnostic and Preventive Medicine, University of Florida, Gainesville, FL 32653, USA; aline.hoffmann@ufl.edu; 5 Department of Veterinary Physiology and Pharmacology, Texas A&M University, College Station, TX 77843, USA; mgolding@cvm.tamu.edu; 6 Department of Atmospheric Sciences, Texas A&M University, College Station, TX 77843, USA

**Keywords:** air pollution, ultrafine particulate matter, developmental toxicology, placenta, gestational exposure, *Nrf2*, oxidative stress, knockout model

## Abstract

Exposure to ultrafine particles (UFPs, PM_0.1_) during pregnancy triggers placental oxidative stress and inflammation, similar to fine PM (PM_2.5_). The *Nrf2* gene encodes a redox-sensitive transcription factor that is a major regulator of antioxidant and anti-inflammatory responses. Disruption of NRF2 is known to substantially enhance PM_2.5_-driven oxidant and inflammatory responses; however, specific responses to UFP exposure, especially during critical windows of susceptibility such as pregnancy, are not fully characterized; To investigate the role of NRF2 in regulating maternal antioxidant defenses and placental responses to UFP exposure, wildtype (WT) and *Nrf2*^−/^^−^ pregnant mice were exposed to either low dose (LD, 100 µg/m^3^) or high dose (HD, 500 µg/m^3^) UFP mixture or filtered air (FA, control) throughout gestation; *Nrf2*^−/^^−^ HD-exposed female offspring exhibited significantly reduced fetal and placental weights. Placental morphology changes appeared most pronounced in *Nrf2*^−/^^−^ LD-exposed offspring of both sexes. Glutathione (GSH) redox analysis revealed significant increases in the GSH/GSSG ratio (reduced/oxidized) in WT female placental tissue exposed to HD in comparison with *Nrf2*^−/^^−^ HD-exposed mice. The expression of inflammatory cytokine genes (*Il1β,* *Tnfα*) was significantly increased in *Nrf2*^−/^^−^ placentas from male and female offspring across all exposure groups. Genes related to bile acid metabolism and transport were differentially altered in *Nrf2*^−/^^−^ mice across sex and exposure groups. Notably, the group with the most marked phenotypic effects (*Nrf2*^−/^^−^ HD-exposed females) corresponded to significantly higher placental *Apoa1 and Apob* expression suggesting a link between placental lipid transport and NRF2 in response to high dose UFP exposure; Disruption of NRF2 exacerbates adverse developmental outcomes in response to high dose UFP exposure in female offspring. Morphological effects in placenta from male and female offspring exposed to low dose UFPs also signify the importance of NRF2 in maternal–fetal response to UFPs.

## 1. Introduction

Ambient particulate matter (PM) represents a significant hazardous element of air pollution [1]. PM is classified as coarse (PM_10_), fine (PM_2.5_), and ultrafine particles (UFPs; PM_0.1_) based on their size [2,3,4]. The fine and ultrafine fractions can penetrate deeper in the airways in comparison with coarse particles, leading to numerous adverse health effects, particularly when exposure occurs during periods of rapid growth and development, such as the prenatal period [5]. Evidence from epidemiological studies links PM exposure, mainly PM_2.5_, during pregnancy with several adverse perinatal outcomes, including preterm birth, infant low birth weight, and placental growth [5,6,7,8,9,10,11].

The placenta is a transient reproductive organ serving as the interface between mother and fetus. There are several functions for the placenta, including nutrient, gas, and waste exchange, which are critical for proper intrauterine growth and development [12,13,14]. Improper placental function can lead to adverse perinatal outcomes [15]. PM exposure affects placental function in many ways, mainly through maternal systemic and placental oxidative stress and inflammation [16]. Additionally, findings from animal exposure models, ex vivo human placental models, and evidence from human placentae demonstrate UFPs can translocate across the placenta, indicating direct exposure [17,18,19,20]. For instance, Wick et al. demonstrated the size-dependent transport of fluorescently labeled particles with diameters of 50, 80, and 240 nm (but not 500 nm) across human placental explants into the fetal circuit [19]. Evidence is emerging on the maternal and fetal-health-related effects specific to UFPs (<100 nm).

UFPs typically exist in high concentrations from traffic sources because of direct emissions and new particle formation [21,22]. Emerging results from our in vivo models demonstrate that UFPs represent an important toxic component driving adverse pregnancy and neonatal pulmonary health outcomes [23,24]. Findings from our research also show that gestational exposure to UFPs alters placental morphology and signaling pathways related to lipid processing, particularly in female offspring [25]. Additionally, other mouse models demonstrate the role of placental oxidative stress in adverse outcomes. For instance, Morales-Rubio et al. verified that gestational UFP exposure increased intrauterine inflammation and oxidative damage, displayed by increased 8-OHdG in mouse placentae [26]. Wang et al. [27] showed increased plasma 8-isoprostane levels, a marker of oxidative stress, in pregnant mice exposed to UFPs. In that model, offspring pulmonary immune maturation was inhibited, playing a role in neonatal respiratory infection risk [28]. Interestingly, findings from a birth cohort study demonstrating increased susceptibility of lower respiratory tract infections in infants prenatally exposed to PM_2.5_ was significantly modified by polymorphisms in the maternal *Nrf2* gene [29].

Nuclear factor E2-related factor 2 (NRF2) is a transcription factor central in response to oxidative stress [30,31]. Under oxidative stress conditions, reactive oxygen species (ROS) promote the dissociation of NRF2 from its repressor protein KEAP1, which typically keeps NRF2 tethered to a ubiquitin ligase complex for degradation [32]. Disassociation from KEAP1 allows NRF2 cytoplasmic accumulation and subsequent binding to antioxidant response elements (AREs) for the transcription of numerous downstream genes. NRF2 binding regulates antioxidant genes such as NAD(P)H quinone oxidoreductase 1 (*Nqo1*), heme-oxygenase 1 (*Ho-1*), superoxide dismutase (*Sod*), catalase (*Cat*), and glutathione peroxidase 1 (*Gpx-1*) [33]. Disruption of NRF2 has been shown to enhance disease susceptibility following a wide range of environmental exposures, including allergic airway inflammatory responses induced by chronic exposure to diesel exhaust PM in an adult mouse model [34]. To date, results from gestational PM exposure models, and specific responses to UFPs relevant to placental and fetal effects are lacking. Therefore, in this study, we investigated the role of NRF2 in maternal systemic and placental responses to gestational UFP exposure. We employed a knockout model (*Nrf2*^−/^^−^) in our established gestational UFP inhalation model [24,25] to further elucidate the underlying mechanisms of UFP toxicity related to developmental endpoints.

## 2. Materials and Methods

### 2.1. Animals and Ultrafine Particle Exposure

All procedures were approved by the Institutional Animal Care and Use Committee of Texas A&M University #2019-0025. *Nrf2*-deficient mice (*Nrf2*^−/^^−^) on C57Bl/6J background were obtained from Dr. Tom Kensler. Genotyping for homozygous wildtype (*Nrf2*^+/+^) and null (*Nrf2*^−/^^−^) mice was carried out as previously described [35] (details in Supplemental Information). Mice were kept under standard housing conditions including 12 h light–dark cycle, 22–24 °C, and 40–60% humidity. Standard 19% protein extruded rodent chow (Teklad Global Diets) and water were given ad libitum, except during exposure timeframes.

To study the role of NRF2 in response to gestational UFP exposure, 8- to 10-week-old female mice were acclimated to filtered air for one-week in exposure chambers. Following acclimation, time-mating, and identification of a vaginal plug, termed gestational day (GD 0.5), wildtype (WT) and *Nrf2*^−/^^−^ pregnant mice were randomly assigned to filtered air (FA) control, low dose (LD; 100 µg/m^3^), or high dose (HD; 500 µg/m^3^) UFP. Particle generation and gestational exposures were conducted as previously described [25]. Gestational exposures occurred for 6 h daily from GD 0.5 to 18.5. Average exposures for LD (100 µg/m^3^) and HD (500 µg/m^3^) equated to a 24 h average of 25 and 125 µg/m^3^, respectively (Figure S1). UFP peak diameter ranged from 71 to 79 nm. Following exposure on GD 18.5, dams were euthanized, and maternal tissues collected and processed. Sex-separated placental and fetal tissues were collected using a dissecting microscope and pooled per litter. Illustration of exposure timeline shown in Figure S2.

### 2.2. Sample Collection and Processing

Post-euthanasia, tissues utilized for histological assessment were fixed in 10% neutral buffered formalin for 24–48 h. Following, tissues were stored in 70% ethanol until trimming, processing, and paraffin embedding. Serial 5 µm cross-sections were obtained where every first and fifth slide were used for hematoxylin and eosin (H&E) staining as observed in Figures 2 and 3. Histological analysis [36] of blinded slides was performed by a board-certified veterinary anatomic pathologist using an Olympus BX53 microscope with an Olympus SC180 camera operating Olympus cellSens 2.3 software. Placenta area of the decidua, spongiotrophoblast, and labyrinth zones were calculated via pixels. For each H&E-stained section, approximately ten 40X random labyrinth zone images were captured to measure fetal vessels and maternal lacunae.

Additionally, tissues were snap-frozen in liquid nitrogen and stored in −80 °C until analysis. Blood and placental collection for redox determination followed Jones and Liang [37]. Briefly, whole blood was added to a borate buffer stock solution containing γ-glutamylglutamate (γ-GluGlut) as an internal standard and centrifuged. The supernatant was transferred to a perchloric/boric acid solution and snap frozen. Samples were thawed and centrifuged to precipitate proteins in preparation for high performance liquid chromatography (HPLC) analysis. Potassium hydroxide/tetraborate solution was used to adjust the pH to 9.0. Dansyl chloride solution was added and placed in the dark for 20–24 h. Finally, chloroform was added, and the perchlorate/chloroform layer was centrifuged where the upper (aqueous) layer was transferred and frozen at −80 °C. Placental tissue homogenates bypassed the first step of borate buffer stock solution and were directly added to the perchloric/boric acid solution. Subsequent processing and analysis steps were the same.

### 2.3. HPLC Run Conditions, Protocol, and Redox Analysis

HPLC run conditions and redox analysis followed Jones and Liang with slight modifications [37]. Briefly, thawed derivatized samples were centrifuged before transfer and 35 µL injection into an UltiMate 3000 HPLC (Thermo Fisher Scientific) autosampler onto a SUPELCOSIL™ LC-NH2 HPLC Column (Supelco 5µm, 4.6 mm × 250 mm) at 35 °C. Fluorescence excitation and emission detection were set at 335.0 and 515.0, respectively, including 48 °C constant temperature. HPLC mobile phases included solvent A (80% v/v methanol/water) and solvent B (acetate-buffered methanol). Flow rate was kept constant at 1 mL per min. Typical mobile phase gradients were as follows: initial conditions of 80% A, 20% B for 10 min; linear gradient change from 10 min to 30 min to 20% A, 80% B; from 35 to 38 min, linear gradient returned to 80% A, 20% B and held until 42 min total run. Approximate elution time frames of compounds of interest were as follows: cystine (CySS) from 9 to 9.5 min; cysteine (Cys) from 10 to 10.5 min; γ-GluGlut from 12 to 13 min; glutathione (GSH) from 19 to 19.5; and glutathione disulfide (GSSG) from 23.5 to 24 min.

### 2.4. RNA Isolation and qRT-PCR

TRIzol reagent was used to extract total RNA from sex-separated, pooled GD 18.5 placentas according to manufacturer’s instructions (Invitrogen, Thermo Fisher Scientific). RNA quantity and purity were assessed using a Nanodrop Spectrophotometer/Fluorometer (DeNovix DS-11 FX+ V3.35) with 260/280 absorbance values ≥1.8. Total RNA was then reverse transcribed into cDNA using Qiagen QuantiTect^®^ Reverse Transcription kit (Cat # 205311). Quantitative real-time PCR was performed with Applied Biosystems™ Power SYBR™ Green PCR Master Mix (Cat # 4367659) on a Roche LightCycler^®^ 96. The reaction conditions were as follows: 50 °C for 2 min; 95 °C for 10 min; and 45 cycles of 94 °C for 15 s, 60 °C for 30 s, and 72 °C for 30 s. Gene transcription levels were analyzed using 2^−ΔΔCT^ method. *Gapdh* was used as the reference gene. Primer sequences are in Table S1.

### 2.5. Statistics

All statistical analyses were performed using GraphPad Prism (V 9.2.0) and expressed using mean ± SD. Phenotypic analyses among groups were tested using two-way analysis of variance (ANOVA) with Tukey’s multiple comparisons test. Tests were considered statistically significant with a *p* value < 0.05.

## 3. Results

### 3.1. Exposure and Particulate Matter Characterization

Our whole-body inhalation exposure system was designed to mimic representative urban UFPs, composed of ammonium sulfate, ammonium nitrate, diesel soot (NIST, SRM 2975), and potassium chloride [24]. A dose response to 6 h daily exposure to 100 and 500 µg/m^3^ UFPs was previously employed by Behlen et al. [25]. This system utilized a differential mobility analyzer coupled with a condensation particle counter, which allowed us to control PM mass concentration in real-time, yielding stable concentrations inside exposure chambers. Figure S1A depicts the average daily exposure by mouse identification number. The randomly assigned pregnant mice were separated into three exposure groups within each genotype: FA, LD, or HD. The average PM mass concentration over the entire exposure time course was 98.14 ± 13.24 (mean ± SD) and 497.68 ± 34.77 µg/m^3^ for LD and HD chambers, respectively. Particle diameters ranged from 0.02 µm to 0.5 µm with 0.071 and 0.079 µm (71 and 79 nm) peak particle diameters for LD and HD chambers, respectively, over the course of gestational exposures (Figure S1C,D). Maternal weight gain was tracked for individual dams, and is shown in Figure S1B for dams with viable pregnancies on GD18.5. These include wildtype (WT) filtered air (FA) control (*n* = 6), WT low dose (LD) (*n* = 6), WT high dose (HD) (*n* = 5), *Nrf2*^−/^^−^ FA (*n* = 3), *Nrf2*^−/^^−^ LD (*n* = 3), and *Nrf2*^−/^^−^ HD (*n* = 5). There were no significant differences in average maternal weight across groups throughout the gestational exposure.

### 3.2. Phenotypic Outcomes Highlight Fetal Weight Impact in Nrf2^−/^^−^ Female HD-Exposed Offspring

**Figure 1:**
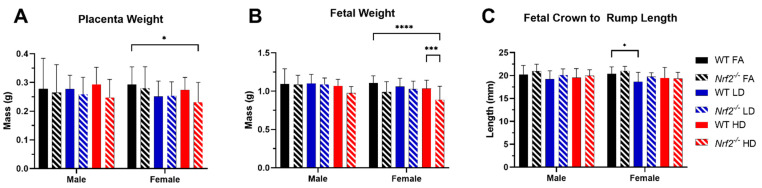
Phenotypic effects of UFPs. (**A**) Average placental weights at GD 18.5 show a significant decrease in the *Nrf2*^−/^^−^ HD female group compared with WT FA control female group. (**B**) Fetal weights at GD 18.5 averaged by sex demonstrates a significant decrease in *Nrf2*^−/^^−^ HD compared with WT HD and WT FA in females. (**C**) Average crown to rump lengths at GD 18.5 show a decrease in the LD versus FA control for WT females. Offspring sample sizes include WT FA male (*n* = 13), WT LD male (*n* = 22), WT HD male (*n* = 20), WT FA female (*n* = 18), WT LD female (*n* = 25), WT HD female (*n* = 24), *Nrf2*^−/^^−^ FA male (*n* = 13), *Nrf2*^−/^^−^ LD male (*n* = 16), *Nrf2*^−/^^−^ HD male (*n* = 23), *Nrf2*^−/^^−^ FA female (*n* = 11), *Nrf2*^−/^^−^ LD female (*n* = 12), and *Nrf2*^−/^^−^ HD female (*n* = 20). Data analyzed using two-way ANOVA with Tukey’s multiple comparison test (* *p* < 0.05; *** *p* < 0.001; **** *p* < 0.0001).

Following exposure on GD 18.5, sex-separated placental and fetal tissues were collected, and phenotypic outcomes assessed. Significant decreases were observed in placental weight in the *Nrf2*^−/^^−^ mice exposed to HD, in comparison with the WT FA group (Figure 1A). The average fetal weight in female *Nrf2*^−/^^−^ offspring was significantly decreased in comparison with the WT FA group and the matched WT HD group (Figure 1B). Interestingly, the only difference noted in fetal crown to rump lengths was in the WT female LD-exposed group (Figure 1C). Offspring sample sizes range from 12–25 per group, collected from 3–6 dams (as indicated in Figure S1 and Figure 1 legends).

### 3.3. Histological Analysis of Placental Tissues Show Impact in Nrf2^−/^^−^ Male and Female LD-Exposed Offspring

**Figure 2:**
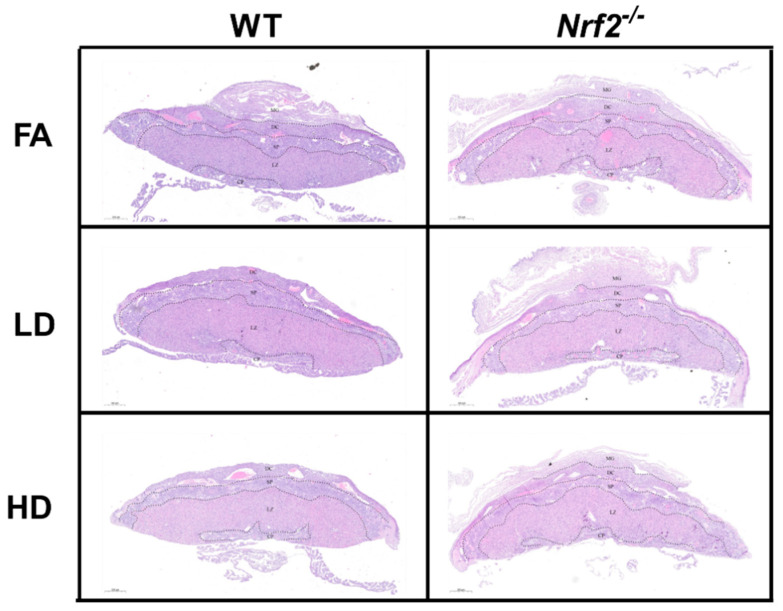
Placental Histology. Representative placental images collected from male offspring depicting wildtype (WT) and *Nrf2*^−/^^−^ mice exposed to filtered air (FA) control, low dose (LD; 100 µg/m^3^), and high dose (HD; 500 µg/m^3^). No gross histologic changes were observed across the different groups. Area measurements were made for decidua (DC), spongiotrophoblast (SP), and labyrinth zone (LZ) layers. Sample sizes for placenta from male offspring: WT FA (*n* = 7), WT LD (*n* = 11), WT HD (*n* = 13), *Nrf2*^−/^^−^ FA (*n* = 10), *Nrf2*^−/^^−^ LD (*n* = 9), *Nrf2*^−/^^−^ HD (*n* = 8).

**Figure 3:**
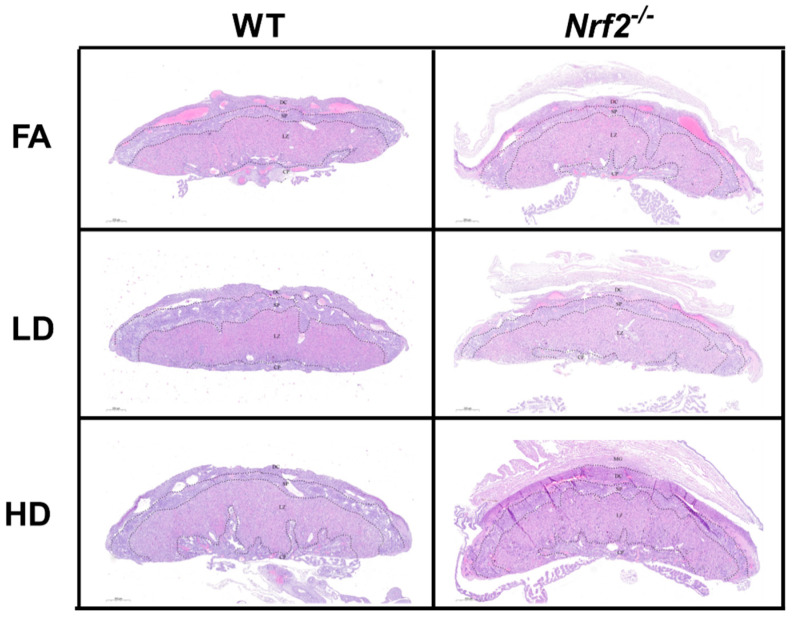
Placental Histology. Representative placental images collected from female offspring depicting wildtype (WT) and *Nrf2*^−/^^−^ mice exposed to filtered air (FA) control, low dose (LD; 100 µg/m^3^), and high dose (HD; 500 µg/m^3^). No gross histologic changes were observed across the different groups. Area measurements were made for decidua (DC), spongiotrophoblast (SP), and labyrinth zone (LZ) layers. Sample sizes for placenta from female offspring: WT FA (*n* = 8), WT LD (*n* = 12), WT HD (*n* = 12), *Nrf2*^−/^^−^ FA (*n* = 5), *Nrf2*^−/^^−^ LD (*n* = 8), *Nrf2*^−/^^−^ HD (*n* = 6).

**Figure 4:**
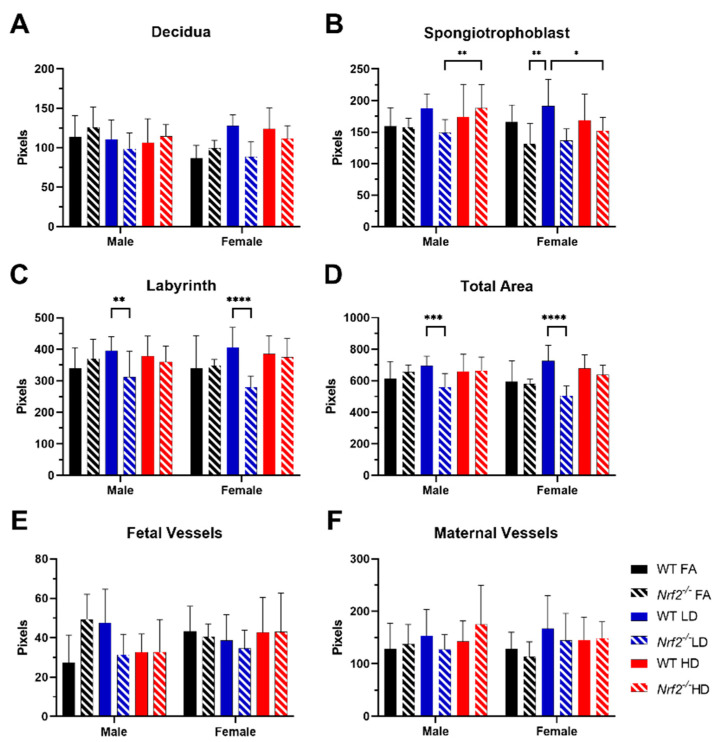
Placental Morphology. Comparisons of WT and *Nrf2*^−/^^−^ placenta from pregnant mice exposed to filtered air (FA) control (black), low dose (LD) (blue), or high dose (HD) (red). Placenta area of the decidua (**A**), spongiotrophoblast (**B**), labyrinth zones (**C**) and total area (**D**) were quantified for each H&E-stained section. Fetal vessels (**E**) and maternal vessels (i.e., lacunae) (**F**) were measured in labyrinth zone images. Error bars represent SD. Data analyzed using one-way ANOVA with Tukey’s multiple comparison test. (* *p* < 0.05; ** *p* < 0.01; *** *p* < 0.001; **** *p* < 0.001). Sample sizes shown in Figure 2 and Figure 3.

Representative placental images are shown for placentas collected from male (Figure 2) and female (Figure 3) offspring in wildtype (WT) and *Nrf2*^−/^^−^ mice exposed to FA, LD, and HD. No gross histological changes were observed across the different groups. Differences between area measurements within different layers of the placenta (decidua, spongiotrophoblast, and labyrinth zones), as well as diameters for the maternal and fetal blood vessels were observed across the different groups (Figure 4). Area measurements for the decidua were significantly reduced in female *Nrf2*^−/^^−^ offspring exposed to LD (4A). For the spongiotrophoblast (4B) and labyrinth (4C) regions, areas were significantly reduced in both male and female *Nrf2*^−/^^−^ offspring exposed to LD. Overall, the total placental areas (4D) were also significantly reduced in these same groups (*Nrf2*^−/^^−^ LD-exposed male and female offspring). Fetal vessel area measurements revealed differing effects of genotype and exposure in placentae collected from male offspring. Fetal vessel areas were greater in the FA-exposed *Nrf2*^−/^^−^ group compared with FA-exposed WT, wherein the LD-exposed *Nrf2*^−/^^−^ group had significantly reduced fetal vessel size as compared with LD-exposed WT (4E). No differences in maternal lacunae sizes were observed between groups (4F).

### 3.4. Oxidative Stress Biomarkers Demonstrate Differential Effect of HD Exposure on GSH and Cys Ratios

**Figure 5:**
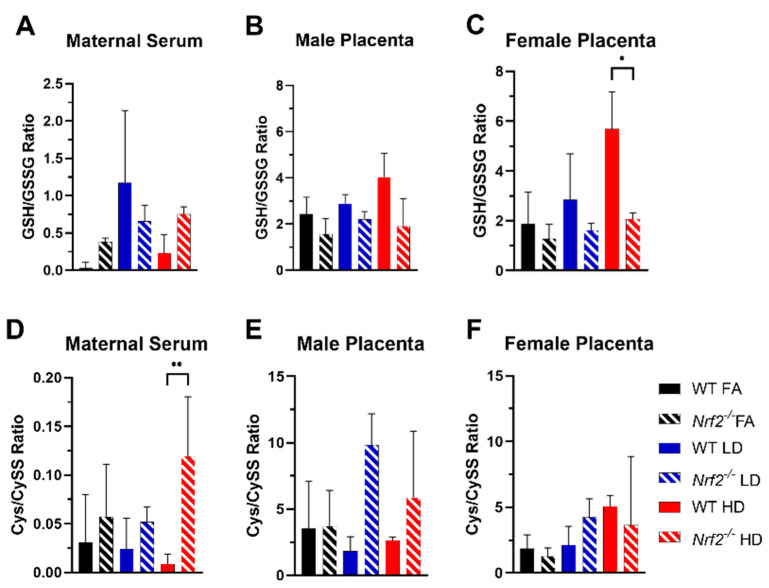
Oxidative stress biomarkers. Glutathione/glutathione disulfide (GSH/GSSG) and cysteine/cystine (Cys/CySS) ratios with comparisons of WT and *Nrf2*^−/^^−^ exposed to filtered air (FA) control (black), low dose (LD) (blue), or high dose (HD) (red) for maternal serum (**A**,**D**) and placenta homogenates from male (**B**,**E**) and female (**C**,**F**) offspring. Error bars represent SD. Data analyzed using one-way ANOVA with Tukey’s multiple comparison test. (* *p* < 0.05; ** *p* < 0.01). Sample sizes for maternal serum: WT FA (*n* = 5), WT LD (*n* = 6), WT HD (*n* = 5), *Nrf2*^−/^^−^ FA (*n* = 3), *Nrf2*^−/^^−^ LD (*n* = 3), *Nrf2*^−/^^−^ HD (*n* = 5). Sample sizes for placenta from male offspring: WT FA (*n* = 6), WT LD (*n* = 11), WT HD (*n* = 6), *Nrf2*^−/^^−^ FA (*n* = 7), *Nrf2*^−/^^−^ LD (*n* = 7), *Nrf2*^−/^^−^ HD (*n* = 15). Sample sizes for placenta from female offspring: WT FA (*n* = 10), WT LD (*n* = 13), WT HD (*n* = 12), *Nrf2*^−/^^−^ FA (*n* = 8), *Nrf2*^−/^^−^ LD (*n* = 4), *Nrf2*^−/^^−^ HD (*n* = 14).

Levels of glutathione (GSH), glutathione disulfide (GSSG), cysteine (Cys), and cystine (CySS) were determined in maternal serum and placenta. Average levels for individual species are shown in Table S2. The ratios of reduced/oxidized species are depicted in Figure 5. GSH is a critical thiol antioxidant that in reduced form (GSH) can donate an electron to detoxify ROS, thus forming the oxidized form (GSSG). A higher ratio of GSH/GSSG indicates a greater percent of reduced GSH available (i.e., enhanced antioxidant capacity). Overall, the GSH/GSSG ratio for female placentas was significantly higher in the WT group exposed to HD, as compared with the HD-exposed *Nrf2*^−/^^−^ group (Figure 5C). Cys is a precursor to GSH synthesis, which forms CySS under oxidative conditions. Interestingly, the Cys/CySS ratio in maternal serum was significantly higher in HD-exposed *Nrf2*^−/^^−^ dams in comparison with HD-exposed WT dams (Figure 5D).

### 3.5. Placental Gene Expression Emphasizes Role of Genotype, Exposure and Sex

**Figure 6:**
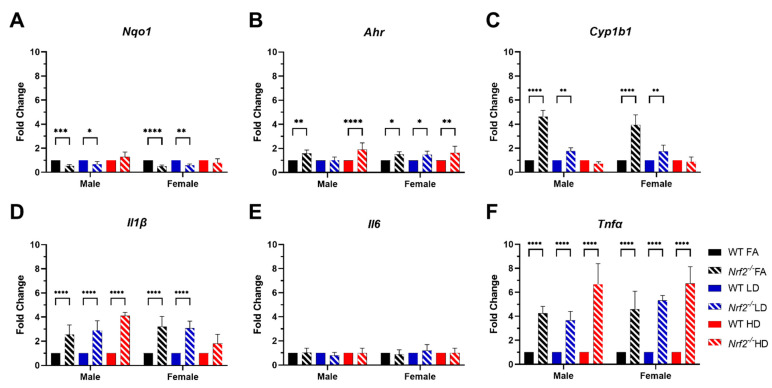
Placental gene expression data. Fold change of *Nqo1* (**A**), *Ahr* (**B**), *Cyp1b1* (**C**), *Il1β* (**D**), *Il6* (**E**) and *Tnfα* (**F**). Error bars represent SD. Data analyzed using two-way ANOVA with Tukey’s multiple comparison test. (* *p* < 0.05; ** *p* < 0.01; *** *p* < 0.001; **** *p* < 0.0001). Male placenta homogenate numbers include WT FA (*n* = 6), WT LD (*n* = 11), WT HD (*n* = 6), *Nrf2*^−/^^−^ FA (*n* = 8), *Nrf2*^−/^^−^ LD (*n* = 7), and *Nrf2*^−/^^−^ HD (*n* = 15). Female placenta homogenate numbers include WT FA (*n* = 10), WT LD (*n* = 13), WT HD (*n* = 12), *Nrf2*^−/^^−^ FA (*n* = 8), *Nrf2*^−/^^−^ LD (*n* = 4), and *Nrf2*^−/^^−^ HD (*n* = 14).

Expression of several genes related to oxidative stress and inflammatory cytokines were assessed in offspring placentas using quantitative real-time PCR. Expression levels of *Nqo1* (Figure 6A) were significantly decreased in *Nrf2*^−/^^−^ FA- and LD-exposed males and females, as expected. We also evaluated *Ahr* (Figure 6B) and found levels were significantly elevated in all *Nrf2*^−/^^−^ groups, except for male LD. The expression of *Cyp1b1* was significantly increased in FA- and LD-exposed *Nrf2*^−/^^−^ groups for both male and female Figure 6C). Strikingly, the expression of inflammatory cytokines *Il1β* and *Tnfα* (Figure 6D,F) was significantly increased in all *Nrf2*^−/^^−^ groups, excluding the female HD group for *Il1β*. *Il6* (Figure 6E) did not demonstrate any significant changes among groups.

**Figure 7:**
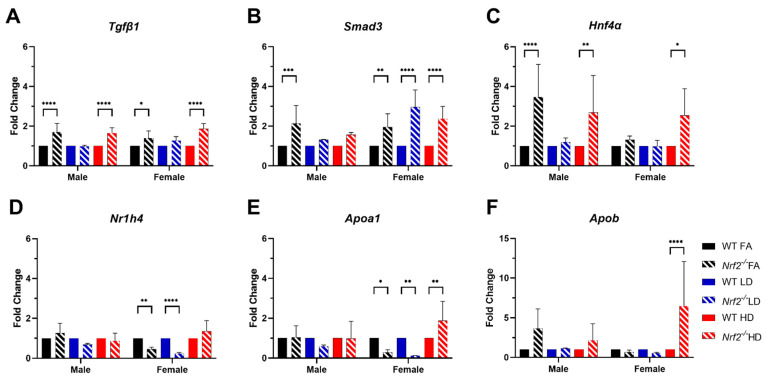
Placental gene expression. Fold change of *Tgfβ1* (**A**), *Smad3* (**B**), *Hnf4α* (**C**), *Nr1h4* (**D**), *Apoa1* (**E**), and *Apob* (**F**). Error bars represent SD. Data analyzed using two-way ANOVA with Tukey’s multiple comparison test. (* *p* < 0.05; ** *p* < 0.01; *** *p* < 0.001; **** *p* < 0.0001). Sample sizes shown in Figure 6.

In our recent work, RNA sequencing of whole placental tissues collected from WT mice exposed to FA, LD, or HD, revealed altered bile acid metabolism [25]. These changes were sex- and dose-specific, with significantly increased expression of several genes in placenta, including *Tgfβ1*, *Smad3*, *Hnf4α*, *Nr1h4* (Farnesoid X Receptor-FXR), *Apoa1*, and *Apob.* To investigate changes in these genes by genotype within each exposure group, we evaluated expression in placental tissues from WT and *Nrf2*^−/^^−^ mice exposed to FA, LD, or HD (Figure 7). *Tgfβ1* expression was significantly increased in *Nrf2*^−/^^−^ mice exposed to FA and HD in male and female placentas, in comparison with WT FA and WT HD, respectively (7A). Similarly, significant increases in *Smad3* across all dose groups were observed in *Nrf2*^−/^^−^ mice for all female groups and FA-exposed *Nrf2*^−/^^−^ males (7B). Expression of *Hnf4α* was also significantly increased in *Nrf2*^−/^^−^ mice in FA- and HD-exposed males, as well as HD-exposed females (7C). *Nr1h4* (FXR) expression was significantly reduced in FA- and LD-exposed *Nrf2*^−/^^−^ females (7D). Expression of *Apoa1* was significantly decreased in female *Nrf2*^−/^^−^ mice exposed to FA and LD, yet increased in *Nrf2*^−/^^−^ HD-exposed females (7E). Likewise, *Apob* expression was significantly increased in *Nrf2*^−/^^−^ HD-exposed females (7D).

## 4. Discussion

Gestational PM exposure is associated with numerous adverse birth outcomes [5]. These include premature birth, fetal growth restriction, and infant low birth weight, all of which are significant risk factors for neonatal morbidity and mortality [6,7,8,38,39]. Although not currently regulated by air quality standards, UFPs may exert enhanced maternal–fetal toxicity due to increased oxidative capacity and their ability to cross the placental barrier, as evidenced in experimental models and human placentae [17,18,19,20]. Findings from experimental models confirm UFP-specific effects on adverse pregnancy outcomes, including reduced gestational length and decreased offspring birth weights and lengths [23,40].

Our previous gestational UFP inhalation model employing either a low dose (LD, 100 µg/m^3^) or high dose (HD, 500 µg/m^3^) throughout gestation demonstrated sex- and dose-specific effects on placental morphology and signaling pathways related to lipid metabolism [25]. In that study, a significant decrease in average placental weights and crown-to-rump lengths was observed in female offspring in the LD exposure group. Moreover, transcriptomic analysis indicated several disturbed cellular functions related to lipid metabolism, which were most pronounced in the LD group, especially in female placental tissue. Building from these findings in WT mice, the main objective of this study was to assess the role of *Nrf2* in placental responses to gestational UFP exposure. Accordingly, we exposed WT or *Nrf2*^−/^^−^ pregnant mice to FA, LD, or HD from GD 0.5 to 18.5. Daily exposure levels were consistently stable. Phenotypic data showed significantly lower fetal crown to rump lengths in WT LD-exposed females, yet placental weights in this group, significantly reduced in our previous model [25], failed to reach statistical significance in this analysis. Our main finding from phenotypic measurements in this study was significantly decreased fetal weights in *Nrf2*^−/^^−^ HD-exposed female offspring. NRF2 signaling is essential in normal placental development and fetal growth, and dysregulation has been implicated in intrauterine growth restriction, preeclampsia, and preterm birth [41]. Mouse models also demonstrate a role of *Nrf2* in trophoblast function in normal placentation and angiogenesis [42]. In our model, *Nrf2*^−/^^−^ mice in the control group, exposed to FA, did not exhibit significantly different phenotypic measures in comparison with the WT FA-exposed group. Likewise, upon challenge with the LD, significant differences were not observed between mice of different genetic backgrounds. The most susceptible group, in terms of effects on fetal weight, was the *Nrf2*^−/^^−^ female mice exposed to the HD. These findings suggest the inability of mice lacking functional NRF2 to respond to an environmental challenge, but only at a high enough dose. The failure of susceptibly to manifest in reduced fetal lengths in this group is unexpected, as only the female WT, LD-exposed group showed significantly lower fetal lengths. However, this may reflect other measures of growth that influence length and are not directly impacted by UFPs in a linear dose–response mechanism. These nuances in different growth measures require further consideration.

In our previous work, WT mice exposed to UFPs throughout gestation exhibited morphological changes in placenta that varied by sex and dose [25]. An increase in placental decidua area, the outer layer on the maternal side, was observed in placenta from female offspring exposed to LD and HD. In our current investigation, we observed decreased decidua areas placenta from female *Nrf2*^−/^^−^ offspring exposed to LD. Moreover, in both sexes of *Nrf2*^−/^^−^ offspring exposed to LD, we observed decreased areas within the spongiotrophoblast and labyrinth layers, intermediary and fetal sides, respectively, as well as overall. These findings suggest placental insufficiency in offspring lacking functional NRF2 largely in both sexes exposed to LD. Indeed, previous studies demonstrate significant reductions in both total and labyrinth volume in placenta of *Nrf2*^−/^^−^ mice [42]. Collectively, these findings highlight the NRF2 deficient signaling may affect nutrient transfer capacity. Additionally, our data demonstrate differential effects of *Nrf2* status and exposure on fetal vessel size. Previously, we reported LD exposure increased fetal vessel size in female placenta, perhaps as a compensatory mechanism. In this study, we saw the lack of *Nrf2* in our FA control group resulted in increased measurement of fetal vessel size in male placenta. Interestingly, male *Nrf2* null mice exposed to LD had decreased fetal vessel size. There were no effects on maternal “vessels,”, i.e., lacunae in *Nrf2* null mice across exposure groups. In our model, as described above, fetal growth effects were most pronounced in HD-exposed female offspring lacking *Nrf2*. The lack of morphological effects in HD-exposed placenta points out the complexity of placental exchange capacity. Future functional measurements may better inform placental nutrient transfer capacity, especially as related to fetal growth restriction.

To further tease apart the effects of UFP exposure in WT and *Nrf2*^−/^^−^ mice, we evaluated oxidative stress biomarkers in maternal serum and placental tissues. Oxidative stress is known to underlie PM-induced adverse pregnancy outcomes [43]. Redox states of GSH/GSSG and Cys/CySS have been applied in many assessments of disease pathologies and environmental exposures [44]. Diesel exhaust particle exposure has been shown to alter Cys redox state in a mouse model of HDM-induced asthma [45]. Likewise, GSH redox was demonstrated to be skewed toward oxidative stress (i.e., decreased GSH/GSSG ratio) in lung cells and neonatal mice exposed to combustion-generated PM with a high free radical content. In our model, we observed a significant increase in the GSH/GSSG ratio in WT, HD-exposed female placenta in comparison with the *Nrf2*^−/^^−^ HD female group. Since several genes related to GSH synthesis have antioxidant response elements (ARE) in their promoter regions, a lack of NRF2 signaling would result in presumably less GSH production [31]. Contrary to other reports showing that PM decreased GSH/GSSG ratios, we noted that HD exposure increased the GSH/GSSG ratio in the female WT HD group. This may indicate HD exposure, which is high at 500 µg/m^3^, triggers NRF2 in response to chronic gestational exposure (GD 0.5 to 18.5) resulting in enhanced GSH production. In this case, *Nrf2*^−/^^−^ female mice exposed to HD fail to mount the same response. Alternatively, in the maternal serum we observed changes in the Cys/CySS ratio, wherein *Nrf2*^−/^^−^ dams exposed to HD had significantly higher ratios compared with WT, HD-exposed dams. GSH and Cys redox states are not in equilibrium, and other models have shown PM-induced oxidative stress impacts Cys redox differently than GSH [45]. Additionally, it is important to note the levels in different compartments, maternal serum versus placental tissue, can reflect unique redox signatures. Additionally, increased maternal Cys levels in plasma have been associated with preeclampsia and adverse pregnancy complications, including premature delivery and low birth weight [46,47]. PM exposure is associated with several of these outcomes. Our data suggest increased CyS/CySS in response to HD PM depends on maternal NRF2 status. Additional measures of oxidative stress, also at varying time points, may further inform our model.

Based on known impacts of UFPs on oxidative stress and inflammatory pathways, as well as recent findings from our gestational UFP exposure model showing how UFPs affect placental bile acid metabolism [25], we evaluated several genes involved in these pathways to investigate the role of NRF2. We observed significantly decreased placental expression of *Nqo1* in *Nrf2*^−/^^−^ male and female FA- and LD-exposed groups, indicating less constitutive activation in *Nrf2*, and failure to elicit response to LD exposure. *Nqo1* is one of two major quinone reductases in mammalian systems and is a prototypical *Nrf2* target gene. Thus, it is expected that we would not see its induction mice lacking *Nrf2*. Other pathways, including the aryl hydrocarbon receptor (AhR) pathway, can influence *Nqo1* transcription. The AhR pathway plays a major role in xenobiotic metabolism, with downstream targets including cytochrome P450s such as CYP1A1 and CYP1B1. PAHs (polycyclic aromatic hydrocarbons) are PM-associated toxicants and known AhR ligands. In our model, all *Nrf2*^−/^^−^ groups, with the exception of LD males, showed increased *Ahr* placental expression. Its downstream target *Cyp1a1* was not detected in any groups (data not shown), and interestingly, *Cyp1b1* expression was significantly increased in *Nrf2*^−/^^−^ FA- and LD-exposed male and female placentae, but not HD groups.

*Nrf2* signaling also plays an anti-inflammatory role via crosstalk with the NF-κB pathway, decreasing IκBα degradation, thereby blocking NF-κB-driven inflammation [48]. Moreover, the induction of heme oxygenase-1 (HO-1) can also inhibit NF-κB signaling and proinflammatory cytokines IL6 and TNFα. Although we did not see differences in *Il6* expression in our model, we did see significantly increased expression of *Tnfα* in all *Nrf2*^−/^^−^ groups, ranging from 4–6-fold, and *Il1β*, ranging from 2–4-fold, in all *Nrf2*^−/^^−^ group, excluding female HD. TNFα is an inflammatory cytokine produced by macrophages/monocytes responsible for a range of signaling events within cells. IL1β is also a potent inflammatory cytokine, and both TNFα and IL1β can drive systemic inflammation. Lack of Nrf2 activity can exacerbate NF-κB signaling, leading to increased cytokine production [49,50]. This trend was somewhat similarly observed for *Tgfβ1* expression. *Tgfβ1* is a multifunctional cytokine, and in our model, expression was significantly increased in male and female *Nrf2*^−/^^−^ placentas in the FA and HD groups. Interestingly, its downstream target, *Smad3*, was increased in all female *Nrf2*^−/^^−^ groups, and only increased in FA male *Nrf2*^−/^^−^ placentas. Collectivity, these data support NRF2 important anti-inflammatory role, basally and in response to an environmental challenge. The lack of functional NRF2 signaling leads to a pro-inflammatory environment in the placenta.

Since gestational UFP exposure was previously shown to impact bile acid metabolism in our mouse model using WT mice [25], particularly in female placentae, we carried out gene expression analysis on keys genes significantly upregulated in our WT exposure model. These included nuclear receptors *Hnf4α* and *Nr1h4* (FXR) and apolipoproteins *Apoa1* and *Apob*, the primary protein components of HDL and LDL, respectively. *Hnf4α* was increased in male *Nrf2*^−/^^−^ FA- and HD-exposed groups, and was also increased in female *Nrf2*^−/^^−^ HD-exposed mice. *Nr1h4* (FXR) gene expression was decreased in female *Nrf2*^−/^^−^ FA and LD groups. Likewise, *Apoa1* was decreased in these groups (female *Nrf2*^−/^^−^ FA and LD). Alternatively, *Apoa1* and *Apob* was significantly increased female *Nrf2*^−/^^−^ HD-exposed mice. Overall, the group with the most marked phenotypic effects (*Nrf2*^−/^^−^ HD-exposed females) corresponded to significantly higher placental *Apoa1* and *Apob* expression suggesting a link between placental lipid dysregulation and placental growth in response to high dose UFP exposure. Our sex-specific findings require additional investigation on the underlying mechanisms, including confirmation of protein expression and functional effects. A previous study assessing *Nrf2* in *Drosophila* flies showed *Nrf2*/Cap-n-collar protein binding occurs in different genetic loci, Hr4 (DHR4) locus versus Hnf4 (dHNF4) locus for females vs. males, respectively [51]. The evidence of female susceptibility in our mammalian model and translation to exposed human populations necessitates further mechanistic study.

## 5. Conclusions

In summary, the disruption of NRF2 directly impacts inflammatory cytokine signaling in placental tissue. The lack of NRF2 exacerbates adverse developmental outcomes in response to UFP exposure, particularly in female offspring exposed to a high dose of UFPs, possibly via oxidative stress and the dysregulation of lipid transport. Other subtle effects of a low dose of UFPs on placental morphology necessitate further study.

## Data Availability

All data is presented in the article.

## Supplementary Materials

The following supporting information can be downloaded at: https://www.mdpi.com/article/10.3390/antiox11020352/s1, Figure S1: Illustration indicating daily exposure, maternal weight gain, and particle diameter distribution, Figure S2: Illustration indicating exposure timeline, Table S1: qRT-PCR primer sequences, Table S2: Average levels of individual redox species, Supplementary Methods: *Nrf2* genotyping protocol.
