# Supplementary material for: NRF2-Dependent Placental Effects Vary by Sex and Dose following Gestational Exposure to Ultrafine Particles
Source: Antioxidants (Basel). 2022 Feb 10;11(2):352. doi: 10.3390/antiox11020352 (PMC8868520; doi:10.3390/antiox11020352)
Supplement: Supplementary file 1 [file antioxidants-11-00352-s001.zip › antioxidants-1488116-supplementary.pdf]

Supplementary Materials for

NRF2-dependent Placental Effects Vary By Sex and Dose following Gestational Exposure to Ultrafine Particles

Jonathan C. Behlen [1], Carmen H. Lau [2], Drew Pendleton [1], Yixin Li [3], Aline Rodrigues Hoffmann [2], Michael C. Golding [4], Renyi Zhang [3,5] and Natalie M. Johnson [1,*]
* Correspondence: nmjohnson@tamu.edu; Tel.: +1-979-436-9325

Supplementary Figures and Captions

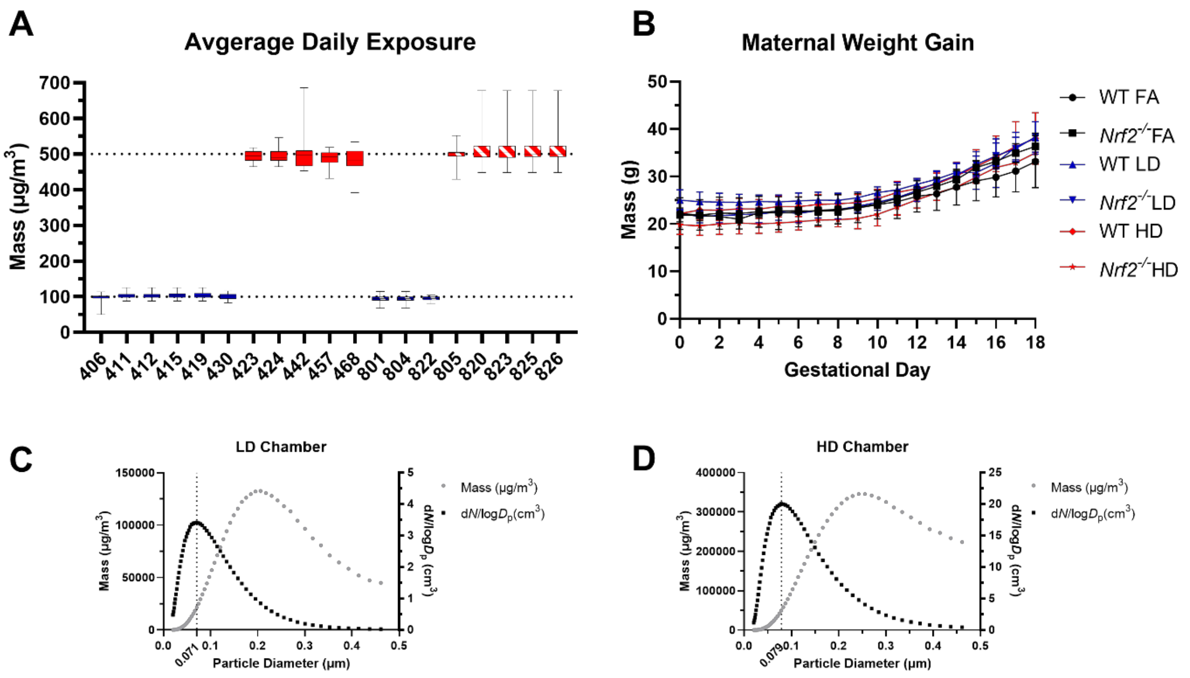

Figure. S1. (A) Individual dams shown by ID number for wildtype (WT) and *Nrf2*$^{-/-}$ pregnant mice with corresponding average daily PM mass concentrations ± SD measured within chambers from GD 0.5 to 18.5. (B) Average maternal weight gain mean ± SD across exposure groups. Groups include wildtype (WT) filtered air (FA) control (n= 6; black line with circle), WT low dose (LD) (n=6; blue line with square), WT high dose (HD) (n=5; red line with triangle), *Nrf2*$^{-/-}$ FA (n=3; black line with inverted triangle), *Nrf2*$^{-/-}$ LD (n=3; blue line with diamond), and *Nrf2*$^{-/-}$ HD (n=5; red line with circle). No significant differences were observed across groups. (C) Low dose (LD) PM size (black) and concentration (gray) distribution, indicating 0.071 μm (71 nm) as the peak particle diameter. (D) High dose (HD) PM particle size (black) and concentration (gray) distribution, indicating 0.079 μm (79 nm) as the peak particle diameter.

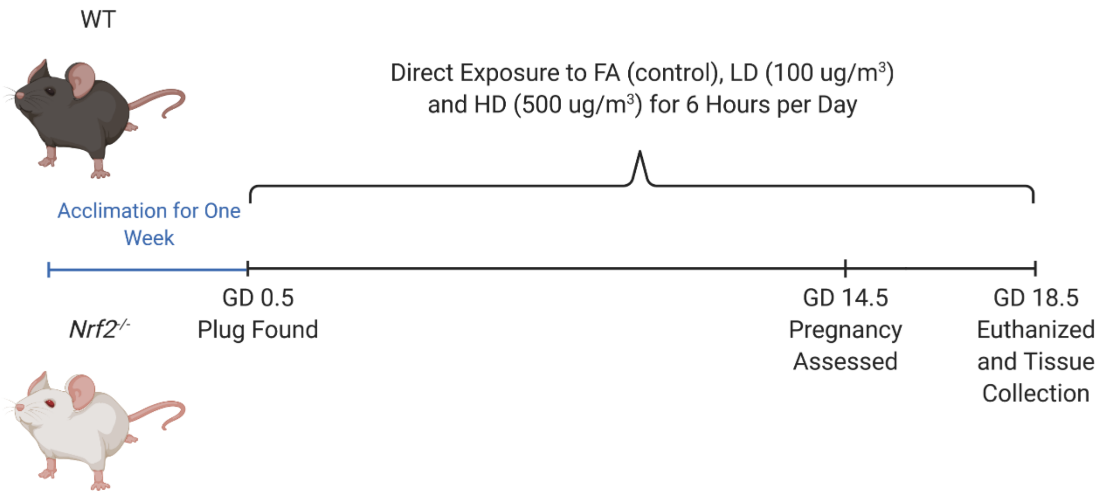

Figure. S2. Illustration indicating exposure timeline. Each dam had a one-week acclimation period to exposure system (without PM) before time-mating. Upon the presence of plug or vaginal cytology with sperm (termed GD 0.5), dams were randomized into an exposure group and exposed throughout gestation until GD 18.5. Created with BioRender.com.

Supplementary Tables

Table S1. Primer Sequences used in qRT-PCR of extracted RNA from pooled GD 18.5 sex-separated placentas.

Target	Forward Primer	Reverse Primer	NCBI Accession Number	Product Size (bp)
Nqo1	TGGCCGAACACAAGAAGCTG	GCTACGAGCACTCTCTCAAACC	NM_008706	112
Ahr	TGTGCAGAATCCCACATCCG	AATCAAGCGTGCATTGGACTG	NM_013464	114
Cyp1b1	CAGTCTGGCGTTCGGTCAC	GCTGCGTTGGATCGAGGAA	NM_009994	197
Il β1	GCCACCTTTTGACAGTGATGAG	AAGGTCCACGGGAAAGACAC	NM_008361	219
Il6	TCGTGGAAATGAGAAAAGAGTTGTG	GGTACTCCAGAAGACCAGAGG	NM_031168	177
Tnf α	CCATGAGCACAGAAAGCATGATC	GCCATTTGGGAACTTCTCATCC	NM_013693	203
Tgfβ1	CAAGGGCTACCATGCCAACT	GTACTGTGTGTCCAGGCTCCAA	NM_011577	67
Smad3	CACGCAGAACGTGAACACC	GGCAGTAGATAACGTGAGGGA	NM_016769	101
Hnf4α	GGTTTAGCCGACAATGTGTGG	TCCCGCTCATTTTGGACAGC	NM_008261	115
Nr1h4	GCTTGATGTGCTACAAAAGCTG	CGTGGTGATGGTTGAATGTCC	NM_001163700	110
Apoa1	GCTCAAGAGCAACCCTACCTT	GCTTTCTCGCCAAGTGTCTTC	NM_009692	75
Apob	AAGCACCTCCGAAAGTACGTG	CTCCAGCTCTACCTTACAGTTGA	NM_009693	111
Gapdh	TGTCAAGCTCATTTCCTGGTATGACA	GAGTTGGGATAGGGCCTCTCTT	NM_001289726	148

Table S2. Levels of individual redox species

	Maternal Serum					
	**WT FA**	***Nrf2*** $^{-/-}$ **FA**	**WT LD**	***Nrf2*** $^{-/-}$ **LD**	**WT HD**	***Nrf2*** $^{-/-}$ **HD**
GSH	0.01 ± 0.03	0.58 ± 0.51	1.64 ± 1.87	1.14 ± 0.22	1.23 ± 1.78	1.25 ± 0.51
GSSG	0.73 ± 0.34	1.49 ± 1.30	1.68 ± 1.08	2.06 ± 0.46	4.92 ± 4.32	1.71 ± 0.77
Cys	0.33 ± 0.47	1.21 ±1.07	0.70 ± 1.22	1.39 ± 0.56	**0.12 ± 0.15**	**1.81 ± 0.29****
CySS	17.73 ± 9.63	15.55 ± 12.82	20.24 ± 8.91	25.47 ± 0.56	16.26 ± 2.64	17.64 ± 6.33
	**Male Placenta**					
	**WT FA**	***Nrf2*** $^{-/-}$ **FA**	**WT LD**	***Nrf2*** $^{-/-}$ **LD**	**WT HD**	***Nrf2*** $^{-/-}$ **HD**
GSH	2.39 ± 2.14	4.29 ± 3.70	**2.57 ± 1.22**	**10.22 ± 1.56****	1.94 ± 0.17	4.13 ± 3.54
GSSG	1.02 ± 0.79	2.51 ± 1.45	**0.86 ± 0.34**	**4.68 ± 0.75****	0.50 ± 0.13	1.91 ± 0.81
Cys	1.90 ± 1.28	1.78 ± 1.29	2.05 ± 1.26	5.00 ± 1.24	1.87 ± 0.29	2.53 ± 2.59
CySS	0.74 ± 9.63	0.49 ± 0.02	1.08 ± 0.25	0.51 ± 0.01	0.71 ± 0.08	0.36 ± 0.13
	**Female Placenta**					
	**WT FA**	***Nrf2*** $^{-/-}$ **FA**	**WT LD**	***Nrf2*** $^{-/-}$ **LD**	**WT HD**	***Nrf2*** $^{-/-}$ **HD**
GSH	2.33 ± 2.26	1.49 ± 1.04	3.68 ± 1.31	4.53 ± 2.69	3.13 ± 0.56	2.89 ± 4.53
GSSG	1.46 ± 0.99	1.36 ± 0.89	1.46 ± 0.40	2.77 ± 1.34	0.60 ± 0.30	1.39 ± 2.18
Cys	1.40 ± 1.60	0.60 ± 0.30	2.41 ± 1.73	1.63 ± 0.57	3.86 ± 0.61	1.35 ± 2.16
CySS	0.60 ± 0.42	0.47 ± 0.05	**1.18 ± 0.24**	**0.38 ± 0.02****	0.76 ± 0.07	0.24 ± 0.14

Oxidative stress biomarker averages ± SD depicting glutathione (GSH), glutathione disulfide (GSSG), cysteine (Cys), and cystine (CySS), values in maternal serum and placenta homogenates across exposure groups and genotype. Sample sizes shown in figure 2. Data analyzed using one-way ANOVA with Tukey's multiple comparison test. (*p<0.05; **p<0.01).

Supplementary Methods

Genotypes of homozygous wild-type and *Nrf2*-deficient mice were confirmed by PCR amplification of genomic DNA extracted from tail snips. PCR amplification was carried out using established methods (Itoh et al. 1997) by using three different primers, 5′-TGGACGGGACTATTGAAGGCTG-3′ (sense for both genotypes), 5′-CGCCTTTTCAGTAGATGGAGG-3′ (antisense for wild type), and 5′-GCGGATTGACCGTAATGGGATAGG-3′ (antisense for LacZ). Conditions were as follows, step 1 95°C 180 sec, step 2 95°C 30 sec, step 3 70°C 30 sec, step 4 72°C 30 sec, repeat steps 2-4 for 35 total cycles, followed by step 5 72°C 120 sec. Wild-type and mutant PCR products detected at 200-300 bp and 400 bp, respectively.

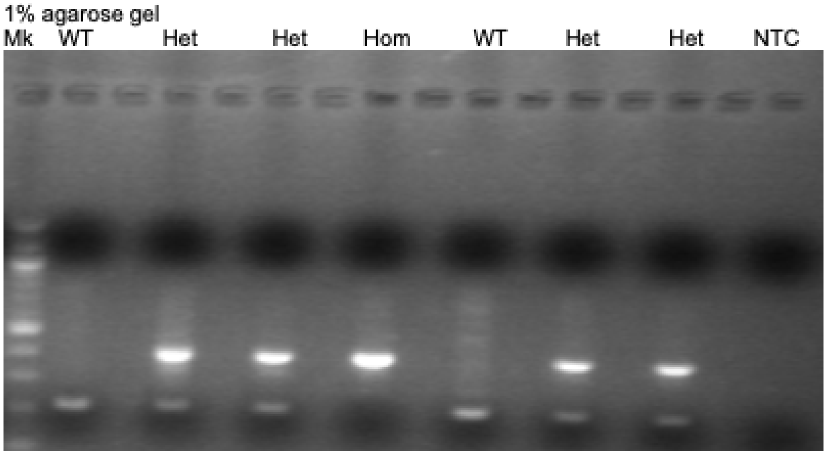

Reference:

Itoh, K., et al., *An Nrf2/Small Maf Heterodimer Mediates the Induction of Phase II Detoxifying Enzyme Genes through Antioxidant Response Elements.* Biochemical and Biophysical Research Communications, 1997. 236(2): p. 313-322.
